# Influence of Oxygen Flow Rate on the Residual Stress, Optical and Morphological Properties of Electron-Beam-Evaporated Tungsten Oxide Thin Films

**DOI:** 10.3390/ma19214471

**Published:** 2026-10-23

**Authors:** Chuen-Lin Tien, Yi-Lin Wang, Shih-Chin Lin, Ching-Chiun Wang

**Affiliations:** 1 Department of Electrical Engineering, Feng Chia University, Taichung 407, Taiwan; br61uce@gmail.com; 2 Mechanical and Systems Research Lab, Industrial Technology Research Institute, Hsinchu 310, Taiwan; shihchin@itri.org.tw (S.-C.L.); juin0306@itri.org.tw (C.-C.W.)

**Keywords:** tungsten oxide, thin films, electron-beam evaporation, oxygen flow rate, optical band gap, surface roughness, residual stress

## Abstract

This study investigates the effect of oxygen flow rate on electron-beam-evaporated tungsten oxide thin films deposited on B270 glass at 100 °C and a nominal deposition rate of 1.0 Å/s. Oxygen flow was varied from 50 to 90 sccm while the other principal deposition conditions were kept constant. The thin films were characterized using optical-constant measurements, UV–Vis spectroscopy, SEM/EDS, a Linnik microscopic interferometer, and a Twyman–Green interferometer. As oxygen flow increased, the refractive index decreased from 1.943 to 1.876 and the extinction coefficient from 4.2 × 10^−3^ to 3.1 × 10^−3^. The measured film thickness varied non-monotonically from 135.8 to 174.1 nm, indicating that the optical-constant trend cannot be explained by thickness variation alone. The apparent optical band gap in the visible region remained within 3.28–3.36 eV. The apparent O/W ratio increased from 2.94 to 3.47, while the apparent surface-feature size increased from approximately 11 to 14 nm and the root-mean-square (RMS) surface roughness increased from 5.03 to 6.03 nm. All films exhibited compressive residual stress, with its magnitude decreasing from 63.7 to 49.5 MPa. These results demonstrate a coordinated oxygen-flow-dependent variation in optical, surface morphological, apparent compositional, and residual stress properties under the electron-beam evaporation technique.

## 1. Introduction

Tungsten oxide is a transition-metal oxide whose optical, electrical, and surface properties are influenced by oxidation state, nonstoichiometric composition, defect-related energy levels, and microstructure. Because these material characteristics can be tuned through processing conditions, tungsten oxide thin films have been widely applied in electrochromic, optical, and gas-sensing fields [1,2,3]. In tungsten oxide thin films, oxygen content, valence-state distribution, and defect structure can further affect optical absorption, carrier behavior, and surface reaction characteristics. Therefore, the deposition atmosphere and film-growth conditions are key factors governing their material properties.

Tungsten oxide thin films can be prepared by a variety of methods, mainly including physical vapor deposition (PVD) and chemical vapor deposition (CVD). Since Deb [4] discovered the electrochromic effect of WO_3_ at room temperature, various methods for preparing tungsten trioxide thin films have been reported in the literature. His first widely recognized report detailed the optical absorption, color-center formation, and electrochromic behavior of thin tungsten oxide films. Svensson et al. [5] reported and discussed electrochromic coatings for potential use on smart windows with dynamic control of radiant energy. Gogova et al. [6,7] investigated the structure of tungsten oxide films obtained by chemical vapor deposition. Among PVD techniques, electron-beam evaporation offers advantages such as controllable deposition rate, high source-material purity, and compatibility with a wide range of materials, and it enables oxide thin films to be prepared at relatively low substrate temperatures [8,9,10]. However, metal oxide materials may undergo compositional deviation or oxygen loss during heating and evaporation under vacuum, causing the vapor species arriving at the substrate surface to have different oxidation states. Consequently, the oxygen supply in the chamber may affect the reoxidation of the deposited species, gas-phase transport collisions, surface nucleation, and packing behavior, thereby modifying the optical and surface properties of the resulting films.

In addition to electron-beam evaporation, other vacuum-based PVD processes have also shown that a reactive oxygen atmosphere can significantly affect tungsten oxide thin films. Ramya Barathy et al. [11] prepared WO_3−x_ films by magnetron sputtering and reported that oxygen conditions altered the porous structure and oxygen-vacancy-related features. Zhu et al. [12] showed that oxygen flow rate, sputtering power, working pressure, and substrate temperature in reactive magnetron sputtering collectively influence the properties of WO_3_ films. Wei et al. [13] varied the oxygen partial pressure and substrate temperature during pulsed laser deposition, thereby establishing correlations between the deposition conditions and the resulting microstructure, stoichiometry, and optoelectronic properties. In addition, reactive-sputtering studies showed that increasing oxygen partial pressure [14] or oxygen concentration [15,16,17,18] can modify film thickness, surface roughness, refractive index, extinction coefficient, and optical transmittance in the visible region of tungsten oxide films, while changes in working pressure also affect particle transport and film properties [19,20]. More recent studies using reactive magnetron sputtering [21], reactive DC sputtering [22,23], radio-frequency magnetron sputtering [24], high-power impulse magnetron sputtering with medium-frequency sputtering [25], and pulsed laser deposition [26] further indicate that discharge mode, oxygen ratio, and deposition energy can simultaneously tune the structure, composition, optical properties, and mechanical behavior of WO_x_ thin films [27]. Film thickness and microstructure are also closely related to the functional performance of tungsten oxide. In 2025, Dwivedi et al. [28] investigated the sputtered-film thickness with defect-related energy levels, optical loss in the visible region, and gas-sensing response. In 2026, Roopa et al. [29] reported that the thickness of thermally oxidized WO_3_ films affects low-temperature NO_2_ sensing behavior and identified a more favorable intermediate thickness range. In addition, the refractive index, extinction coefficient, and optical absorption of tungsten oxide are influenced by material composition and film microstructure. Therefore, to establish a high-quality WO_x_ thin film process, it is necessary to consider not only optical properties but also surface morphology, elemental composition, film thickness and mechanical stress state.

Previous studies have explored the effects of oxygen partial pressure, oxygen ratio, operating pressure, film thickness, and deposition energy on the properties of WO_x_ films. However, under the condition that other initial deposition conditions are fixed, there is still a lack of systematic process-performance studies that link oxygen flow rate with parameters such as optical constants, UV-Vis absorption, apparent elemental composition, nanoscale surface roughness, and residual stress for tungsten oxide (WO_x_) films prepared by electron-beam evaporation. This gap is important because oxygen flow can simultaneously modify the surface-growth environment, apparent oxygen signal, optical loss, surface morphology, and stress relaxation. The present study therefore adopts an integrated characterization strategy rather than treating optical, surface, and mechanical properties independently. The main research question is whether a controlled change in oxygen flow rate produces a coherent, experimentally observable tuning of multiple tungsten oxide film properties even when the actual film thickness does not vary monotonically. In this work, the novelty of the present work lies in establishing a process–property relationship for electron-beam-evaporated WO_x_ films using independent optical, morphological, compositional, surface roughness, and residual stress measurements under a common deposition-condition matrix. Tungsten oxide thin films were deposited by electron-beam evaporation at a deposition temperature of 100 °C and a nominal deposition rate of 1.0 Å/s, while the oxygen flow rate was set to 50, 60, 70, 80, and 90 sccm. A self-built ellipsometer was used to measure the refractive index and extinction coefficient at 650 nm, while UV–Vis spectroscopy and Tauc-plot analysis were used to evaluate optical absorption and the apparent optical band gap in the visible region. FE-SEM/EDS, a Linnik microscopic interferometer, and a Twyman–Green interferometer were further employed to evaluate surface morphology, apparent elemental composition, surface roughness, and residual stress, respectively. The resulting dataset is used to identify which film properties exhibit systematic oxygen-flow dependence and which remain comparatively insensitive within the investigated process window. The novelty of this study lies in establishing an experimentally measured multi-property process–property correlation for electron-beam-evaporated tungsten oxide films under a controlled oxygen flow rate. In contrast to the extant body of literature addressing single properties or deposition technologies, the present study undertakes a multifaceted evaluation encompassing optical constants, UV-Vis absorption, apparent EDS composition, nanoscale surface morphology, RMS surface roughness, and residual stress in tungsten oxide films.

## 2. Materials and Methods

### 2.1. Coating Substrates and Electron-Beam Evaporation

B270 glass substrates were mainly used for optical, surface-roughness, and residual-stress measurements, whereas Si substrates were mainly used for SEM/EDS analysis to reduce charging effects and facilitate observation of surface morphology and elemental signals. Before deposition, the substrates were degreased with alcohol and ultrasonically cleaned to remove particles and contaminants. Surface plasma treatment was then applied to further improve substrate cleanliness and film adhesion stability.

WO_x_ thin films were prepared using a high vacuum electron-beam evaporation system (Showa Shinku Co., Ltd., Kanagawa, Japan).

The deposition temperature was fixed at 100 °C, the nominal deposition rate was maintained at 1.0 Å/s, and the target thickness was 150 nm. The base pressure was approximately 7.0 × 10^−4^ Pa. The source material for thin film deposition was WO_3_ granule (99.99% purity). During film deposition, the working pressure was 2.2–3.4 × 10^−2^ Pa for different oxygen flow rates. Oxygen gas was introduced using an automatic pressure control system, with flow rates set to 50, 60, 70, 80, and 90 sccm. Except for oxygen flow rate, all major process conditions were kept constant to compare the effects of oxygen flow on the film properties, as shown in Table 1.

### 2.2. Optical Constants and UV–Vis Measurements

The refractive index n and extinction coefficient k were measured using a self-built simplified ellipsometer with a polarizer–sample–analyzer configuration [30,31]. A 650 nm semiconductor laser was used as the light source, and the angles of incidence were set to 30° and 60°. At each incidence angle, the reflected-light intensity was acquired by varying the polarizer and analyzer angles, and n and k values were then calculated by the Drude model and numerical iteration. Because this system is a simplified fixed-wavelength configuration, the resulting optical constants were used primarily to compare the relative variations among samples deposited at different oxygen flow rates at a wavelength of 650 nm.

The Drude model and numerical iteration with a Matlab program (R2024b) were employed to obtain n and k. The ellipsometer calibration process was included. Prior to each measurement, the uncoated silicon substrate and a SiO_2_ calibration film of known thickness were examined using a reference sample. The light intensity of measured thin films was detected with two incidence angles of 30° and 60° by applying the polarizer–sample–analyzer configuration. The values of light intensity were then converted into ellipsometric parameters. These parameters were then entered into the Drude model conversion formula in order to calculate the optical constants of the sample using Matlab software. The standard deviations of repeated measurements were found to be ±0.002–0.006 for n and ±0.1–0.2 × 10^−3^ for k.

Thin film thickness was measured using a KLA Tencor α-step 500 surface profilometer. During deposition, part of each substrate was masked to create a step between the coated and uncoated regions, and the actual film thickness was obtained from the measured step height. UV–Vis transmittance and absorbance spectra were recorded using a Shimadzu UV-2600i spectrophotometer, with uncoated B270 glass used as the reference, to compare the optical responses of the tungsten oxide thin films deposited at different oxygen flow rates.

### 2.3. Apparent Optical Band-Gap Analysis

The absorption coefficient (α) of each film was calculated from the measured absorbance (A) and the actual film thickness (d) according to:

α = 2.303A/d.(1)

The optical loss in the visible region was analyzed using the Tauc relation [32,33].

(αhν)^m^ = B(hν − E_g_) (2)

An indirect allowed transition was assumed in this study; therefore, (αhν)^1^⁄^2^ was plotted as a function of hν. When calculating α, the film thickness d was converted to centimeters so that α was obtained in cm^−1^.

The absorption edge of the tungsten oxide films was analyzed using an indirect allowed transition model. A plot of (αhν)^1^⁄^2^ versus photon energy hν was constructed, and a linear region near the absorption edge was fitted and extrapolated to the horizontal axis to obtain the Tauc-derived apparent optical band gap in the visible region Eg. Because the Tauc extrapolation can be influenced by film thickness, defect states, the Urbach tail, and the selected linear region, Eg is treated here as a relative comparison parameter among the different process conditions.

### 2.4. Surface Morphology and EDS Elemental Composition

Surface morphology was observed using a Hitachi S-4800 field-emission scanning electron microscope (FE-SEM; Hitachi High-Tech, Tokyo, Japan). Before SEM observation, the samples were coated with a thin Pt conductive layer using a HITACHI E-1010 ion sputter coater (Minato-ku, Tokyo, Japan). All SEM images were acquired at a magnification of 200,000× with a scale bar of 200 nm. Surface particle sizes were preliminarily estimated from the image scale. Because the images may include particle agglomeration, Pt-coating effects, and differences in surface contrast, the reported values are referred to as “apparent particle size” rather than crystallographic grain size.

Elemental analysis was performed using energy-dispersive X-ray spectroscopy (EDS; Bruker XFlash 6130, Billerica, MA, USA). The EDS acquisition conditions (15 kV, Pt-coated samples) and Pt-coating contributions to the measured O and W signals were considered. The O and W signals were normalized to calculate the apparent O/W atomic ratio and compare relative changes in oxygen content among samples deposited at different oxygen flow rates. Because EDS quantification of light elements in nanoscale oxides is semiquantitative and may be affected by electron-beam interaction depth, substrate signals, surface adsorption, and the Pt conductive coating, the EDS-derived O/W ratio is not treated as an exact stoichiometric ratio and is not used to directly infer the oxygen-vacancy concentration.

Surface roughness was measured using a self-built Linnik microscopic interferometer [34]. A 632.8 nm He–Ne laser was used as the light source together with a 50× objective lens with a numerical aperture of 0.55, and the measurement area was 50 μm × 50 μm. The reconstructed surface-height data were used to calculate the root-mean-square (RMS) roughness (Rq) and arithmetic mean roughness (Ra), enabling the comparison of the effects of oxygen flow rate on the degree of surface height variation.

### 2.5. Residual-Stress Measurement

Residual stress was measured using a modified Twyman–Green interferometer. A 632.8 nm He–Ne laser and a CCD camera were used to record interference fringes before and after film deposition. Fourier filtering, phase extraction, and phase unwrapping were performed in MATLAB to reconstruct the substrate surface profile and radius of curvature. The change in substrate curvature before and after deposition was then substituted into the Stoney equation to calculate the residual stress of the film [35,36]:

(3) $$\sigma_{f}=\frac{E_{s}t_{s}^{2}}{6\left(1-\nu_{s}\right)t_{f}}\left(\frac{1}{R}-\frac{1}{R_{0}}\right)$$

where *σ_f_* is the residual stress in thin films; Es and νs are the Young’s modulus and Poisson’s ratio of the substrate, respectively; t_s_ and t_f_ are the substrate and film thicknesses, respectively; and R_0_ and R are the radii of curvature before and after deposition, respectively. In this study, negative values denote compressive residual stress, whereas positive values denote tensile stress.

## 3. Results

### 3.1. Optical Constants and Film Thickness

**Figure 1:**
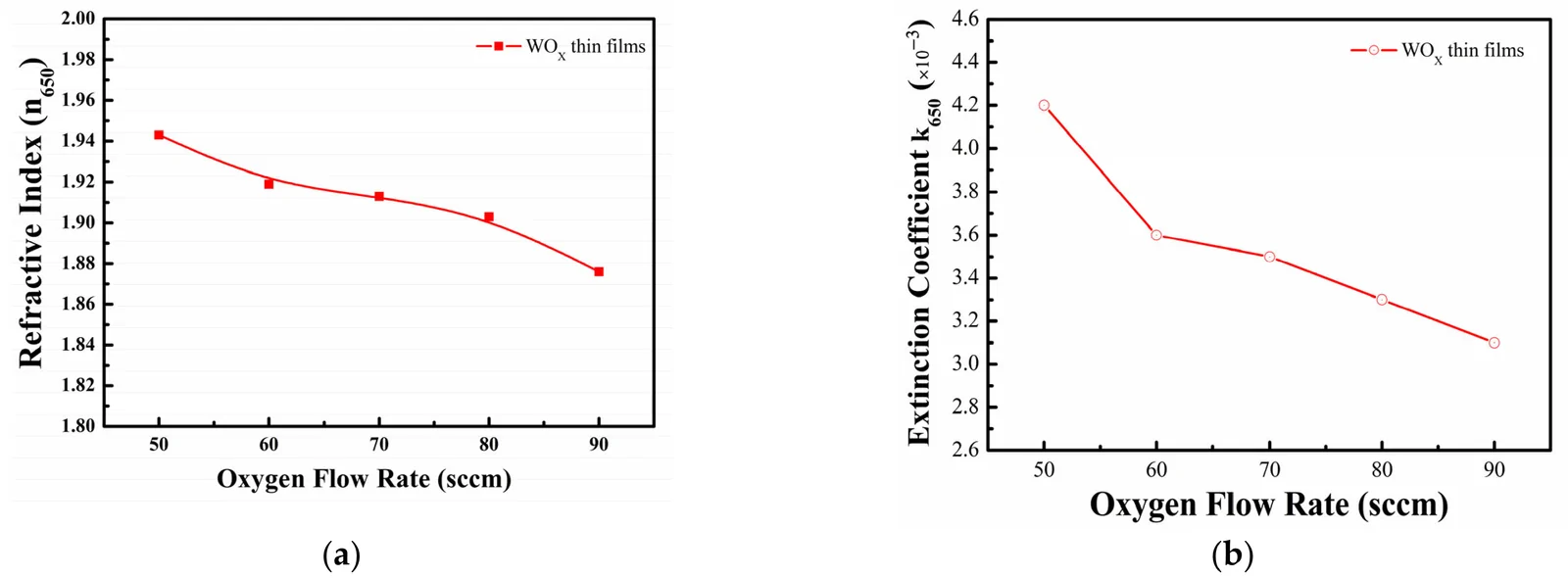
Optical constants of WO_x_ thin films at 650 nm as a function of oxygen flow rate: (**a**) refractive index n_650_; (**b**) extinction coefficient k_650_.

The optical constants measured at wavelength of 650 nm are summarized in Table 2 and Figure 1. As the oxygen flow rate increased from 50 to 90 sccm, the refractive index n_650_ continuously decreased from 1.943 to 1.876, and the extinction coefficient k_650_ showed the same trend, decreasing from 4.2 × 10^−3^ to 3.1 × 10^−3^. In contrast, the actual film thickness did not vary monotonically, with measured values of 135.77, 174.10, 166.70, 163.25, and 164.44 nm, respectively. This measurement yields a thickness uncertainty within ±1 nm.

Because the thin film thickness varied non-monotonically with oxygen flow rate, the monotonic decreases in n_650_ and k_650_ cannot be attributed solely to a thickness effect. This decoupling between thickness and optical-constant trends is an important result of the present study: although the deposition process was nominally maintained at a constant deposition rate, the oxygen-flow condition produced a non-monotonic thickness response while n_650_ and k_650_ changed monotonically. The optical response should therefore be interpreted in relation to oxygen-flow-dependent changes in apparent oxygen signal, film-packing state, and defect-related absorption. These trends predict atomic-scale variations in surface morphology and packing density during thin film formation. However, current measurement datasets can only identify macroscopic features of these variations to understand the impact of oxygen flux changes on tungsten oxide film properties, rather than delving into the specific atomic-scale mechanisms leading to these variations.

### 3.2. UV–Vis Spectra and Optical Loss in the Visible Region

**Figure 2:**
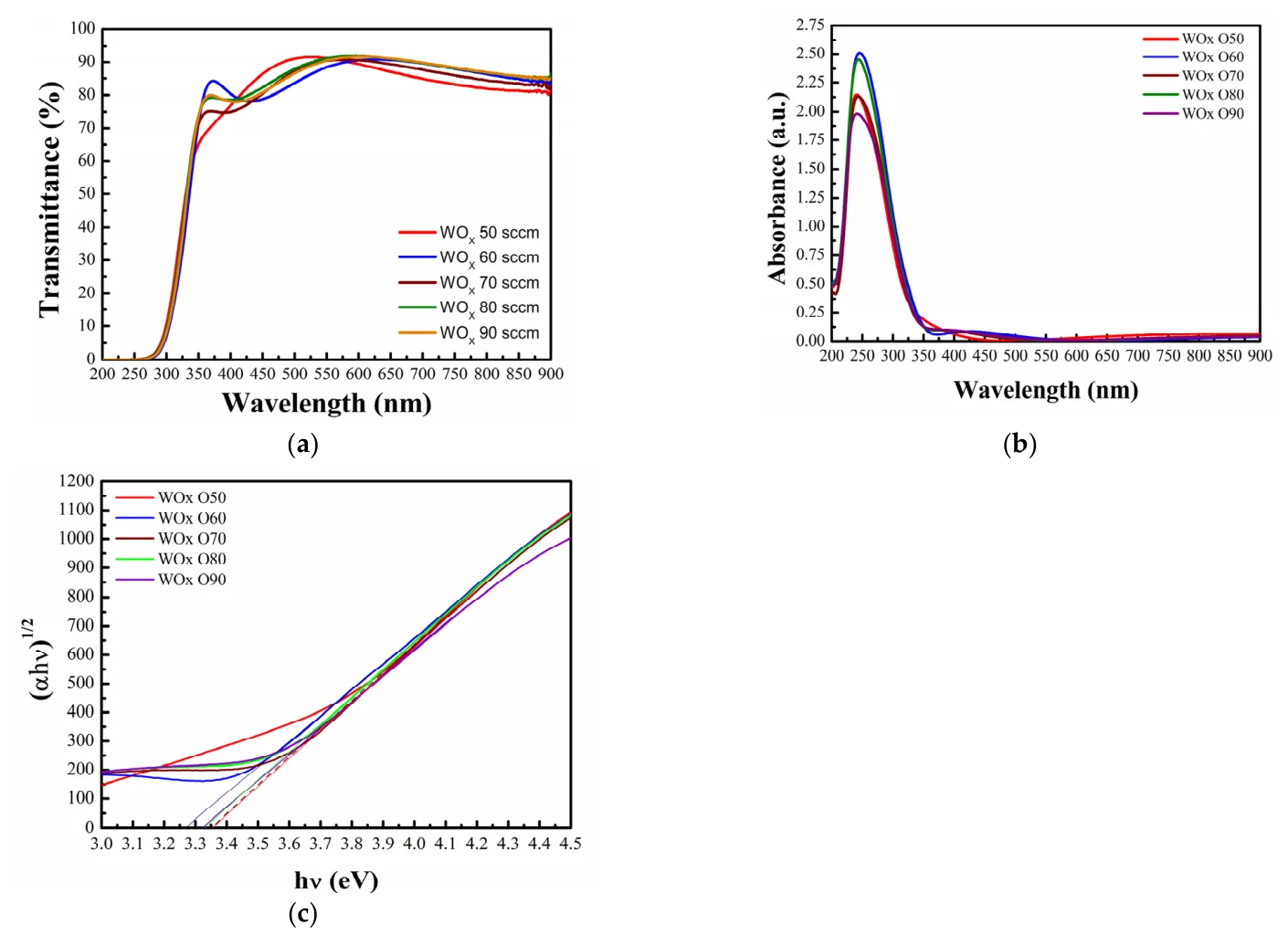
Optical spectra of WO_x_ thin films: (**a**) UV–Vis transmittance spectra including the uncoated B270 reference; (**b**) absorbance spectra; (**c**) indirect-transition Tauc plots used to estimate the apparent optical band gap in the visible region.

**Figure 3:**
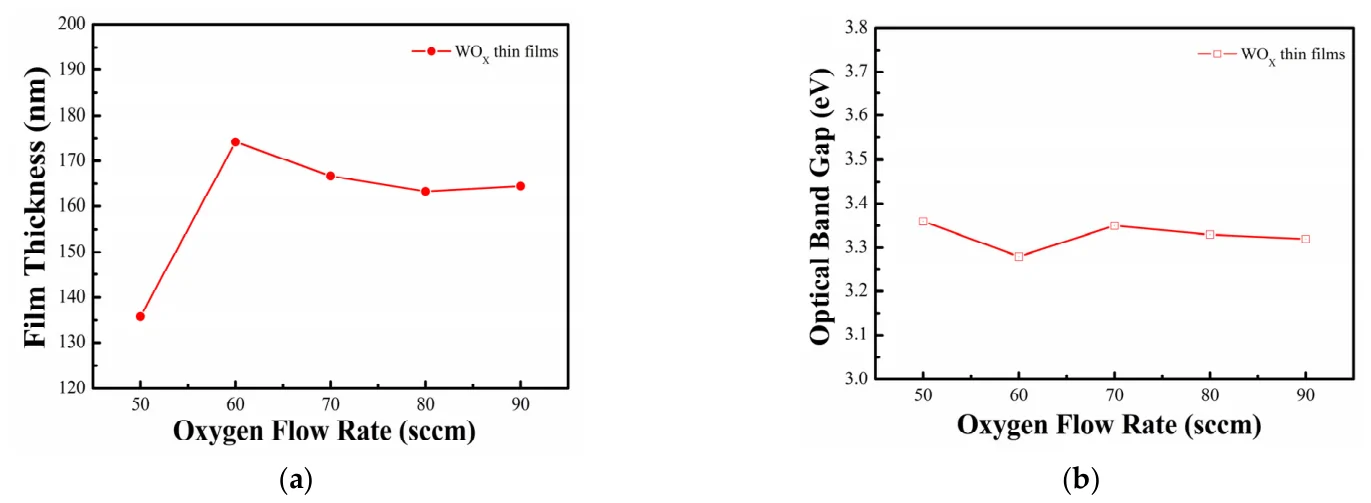
(**a**) Thin film thickness and (**b**) Tauc-derived optical band gap in the visible region of WO_x_ thin films as a function of oxygen flow rate.

Figure 2a shows the UV–Vis transmittance spectra of the WO_x_/B270 samples together with the uncoated B270 reference substrate. All coated samples exhibited very low transmittance in the deep-ultraviolet region, followed by a rapid increase over approximately 300–370 nm, as shown in Figure 2a. In the visible region, all WO_x_ films showed good transparency, with only limited differences among the transmittance curves at different oxygen flow rates. The absorbance spectra in Figure 2b show pronounced ultraviolet absorption, with relatively high absorbance around 240–260 nm followed by a rapid decrease beyond approximately 300–350 nm; absorbance remained low across most of the visible region. Changes in oxygen flow rate mainly produced subtle differences in ultraviolet absorption intensity and absorption-edge position, which may be jointly influenced by oxidation state, defect states, film thickness, and the optical response of the substrate. Figure 2c shows the Tauc plots obtained using the indirect allowed transition model. Linear extrapolation yielded E_g_ values of 3.36, 3.28, 3.35, 3.33, and 3.32 eV at 50, 60, 70, 80, and 90 sccm, respectively. Thus, E_g_ varied only within the narrow range of 3.28–3.36 eV and followed a non-monotonic trend, with the 60 sccm sample showing the lowest extrapolated optical band gap. Figure 3a shows the variation of WO_x_ film thickness with oxygen flow rate, and Figure 3b shows the variation of the optical bandgap derived by Tauc in the visible region with oxygen flow rate.

### 3.3. Surface Morphology and Apparent Elemental Composition

**Figure 4:**
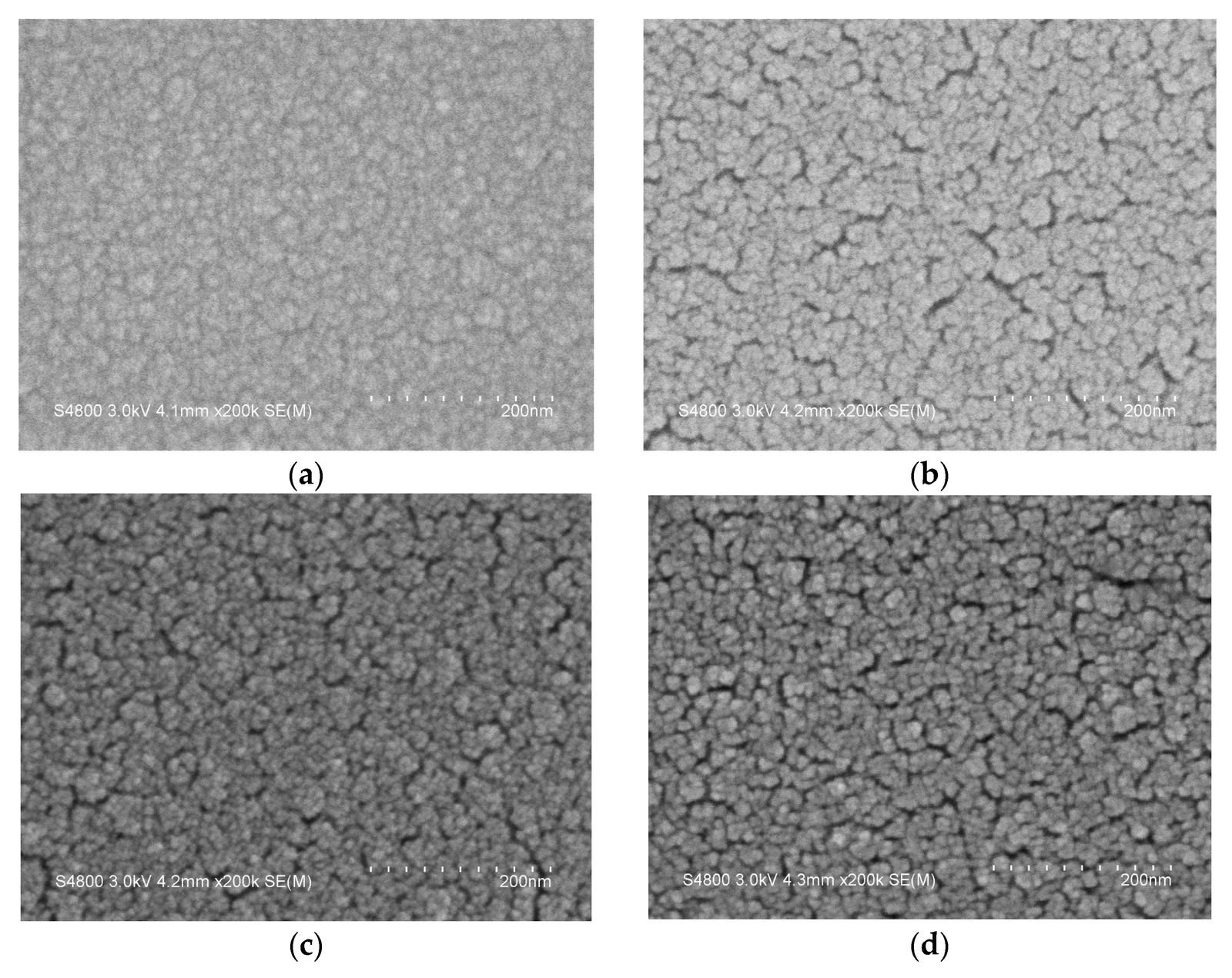

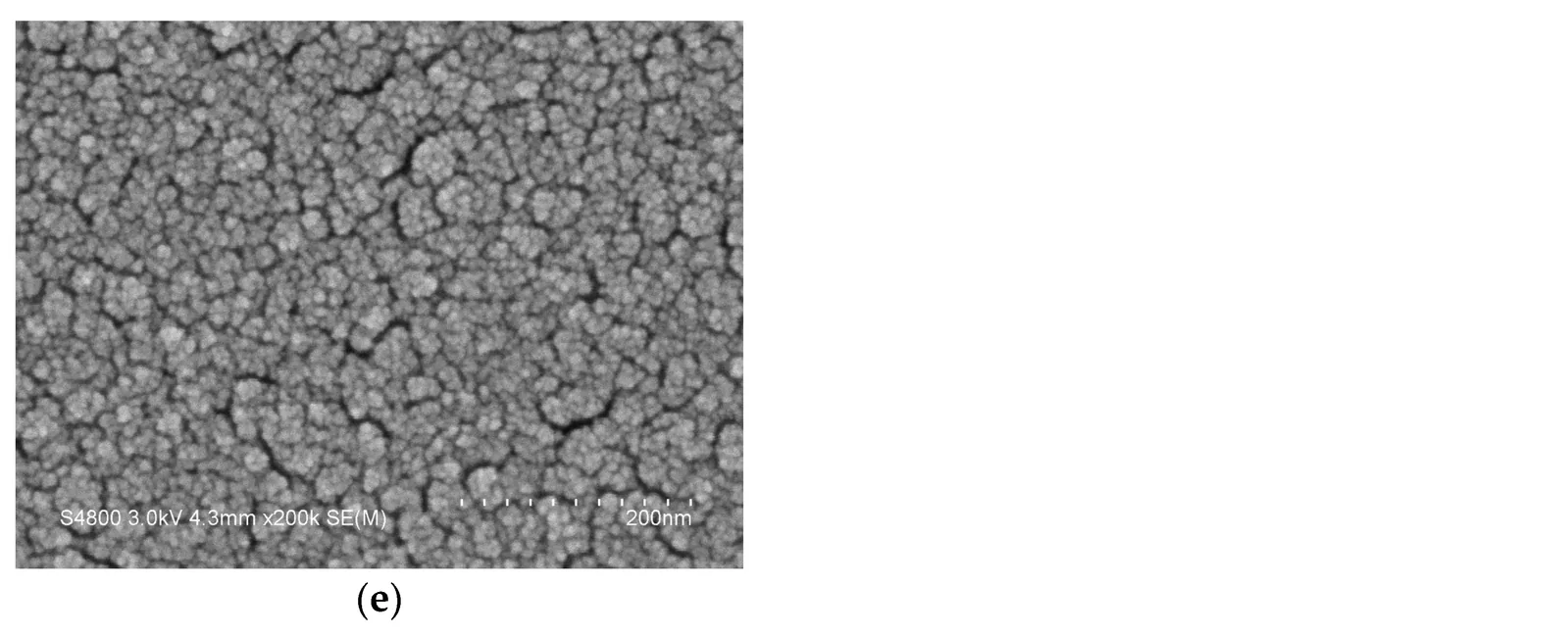
SEM surface images of WO_x_ thin films deposited at oxygen flow rates of (**a**) 50, (**b**) 60, (**c**) 70, (**d**) 80, and (**e**) 90 sccm. The original SEM magnification was 200,000×, and the scale bar was 200 nm.

Figure 4 shows SEM surface images of the WO_x_ thin films deposited at different oxygen flow rates. All samples formed continuous nanogranular surfaces without obvious large-area cracks or delamination. The 50 sccm sample exhibited finer and relatively uniform surface features. As the oxygen flow rate increased to 60 and 70 sccm, particle boundaries and local surface undulations became more apparent. At 80 and 90 sccm, more pronounced particle boundaries and localized agglomeration were observed. The apparent particle size preliminarily estimated from the image scale increased from approximately 11 to 14 nm, indicating a slight increase in surface particle/agglomerate features with increasing oxygen flow rate.

**Figure 5:**
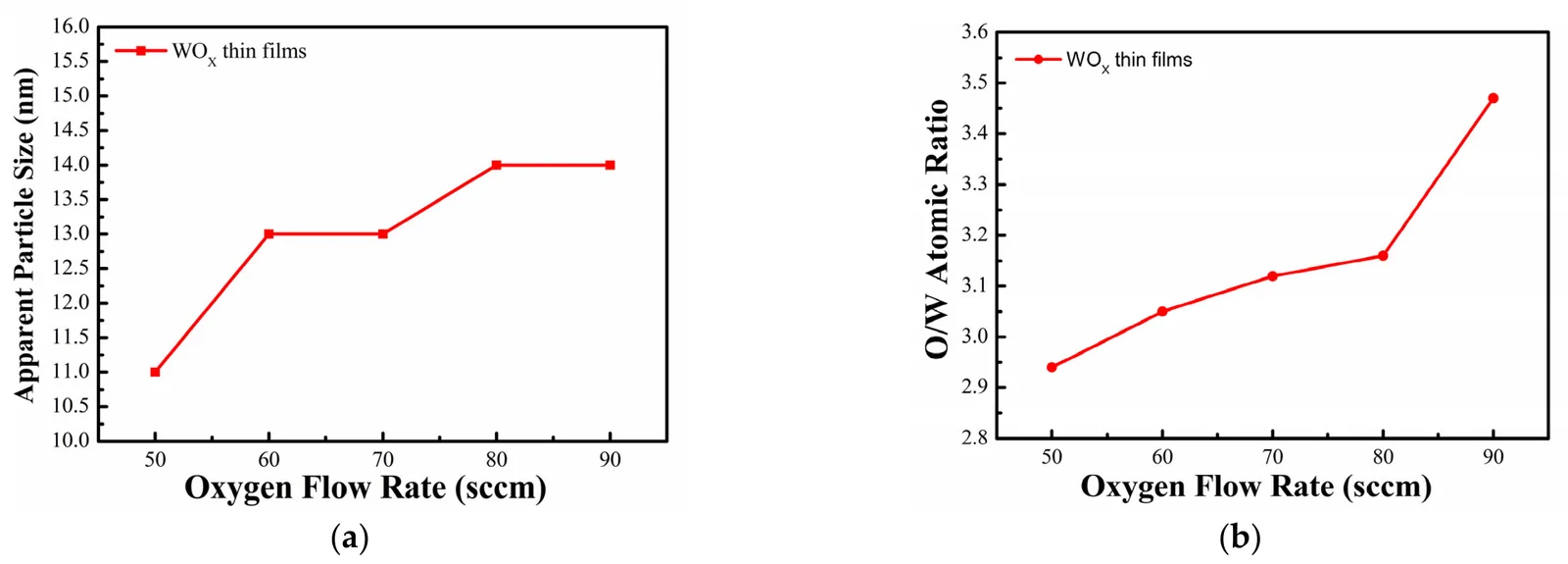
Surface-morphology and elemental-composition parameters of WO_x_ thin films deposited at different oxygen flow rates: (**a**) particle size estimated from SEM images; (**b**) O/W atomic ratio obtained from EDS.

The EDS detected W and O as the primary film-related elements. As shown in Table 3, when the oxygen flow rate increased from 50 to 90 sccm, the normalized O atomic percentage increased from 74.59 to 77.63 at.%, whereas the W atomic percentage decreased from 25.41 to 22.37 at.%. Accordingly, the apparent O/W atomic ratio increased from 2.94 to 3.47 overall. This result supports a relative increase in the apparent oxygen signal with increasing oxygen supply; however, it should not be interpreted as evidence of an exact oxygen-rich stoichiometric composition. To investigate the relationship between the surface morphology and elemental composition parameters of WOx thin films deposited under different oxygen flow rates, the results are shown in Figure 5. Figure 5a shows the particle size estimated from SEM images. Figure 5b shows the O/W atomic ratio obtained from EDS.

### 3.4. Surface Roughness

**Figure 6:**
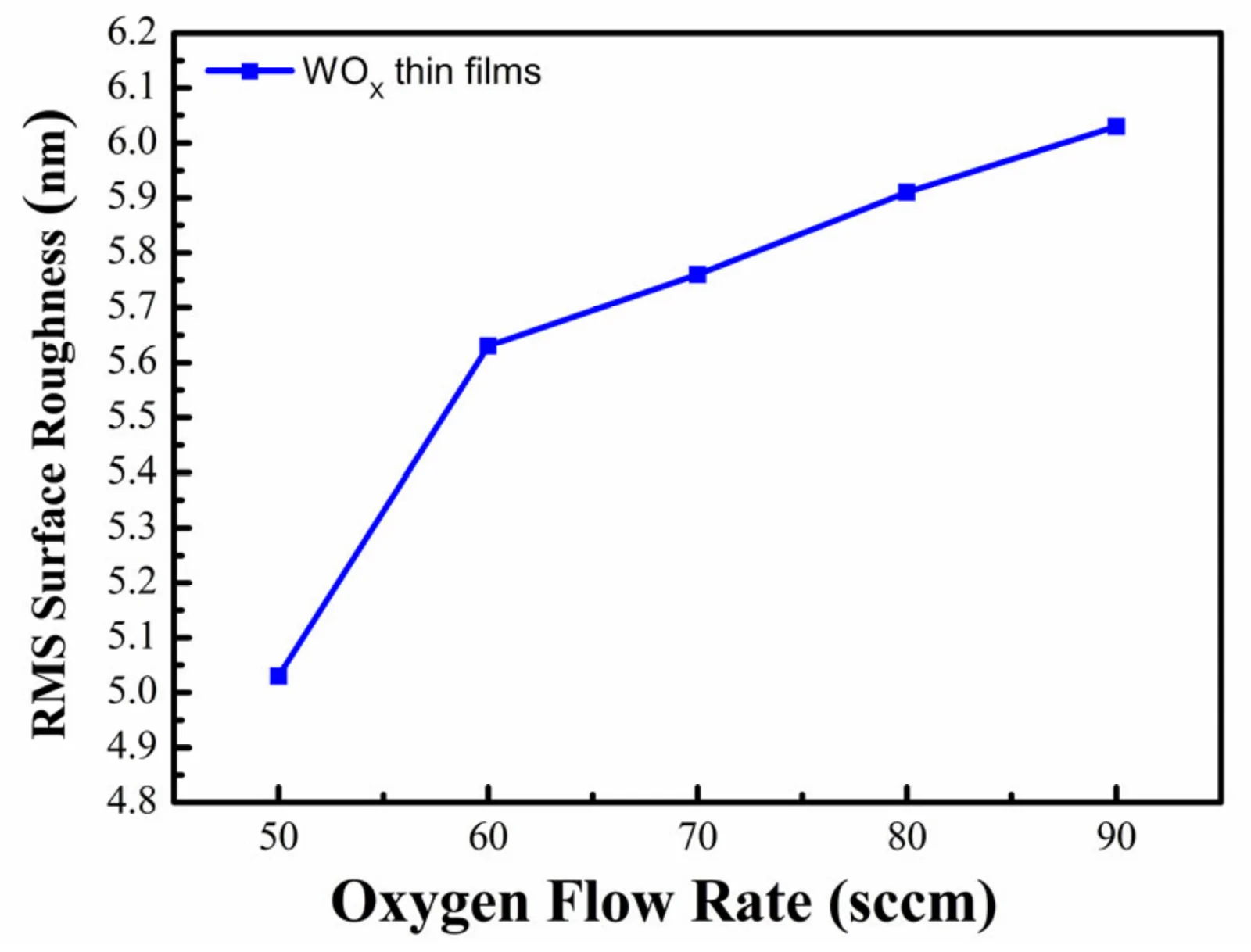
Root-mean-square roughness of WO_x_ thin films deposited at different oxygen flow rates.

**Figure 7:**
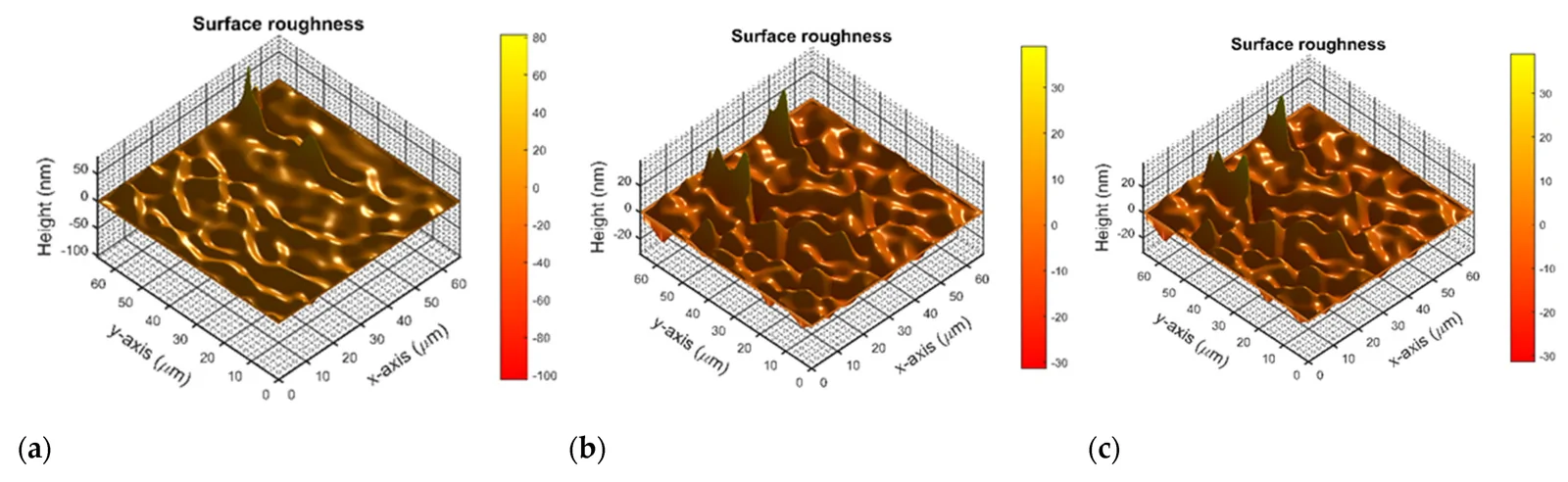

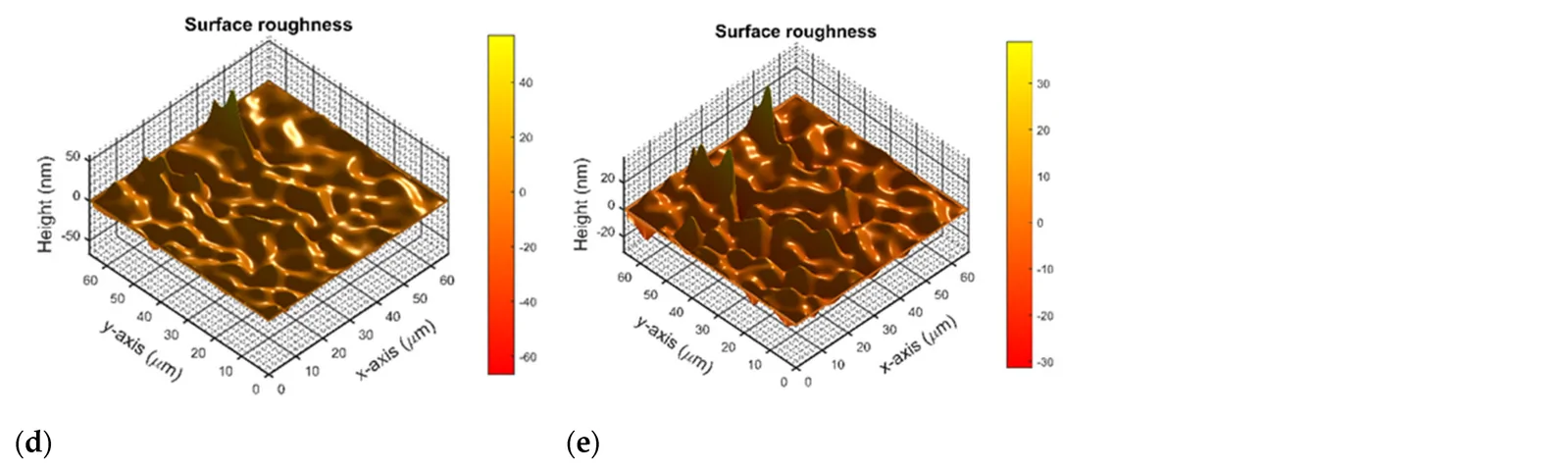
Three-dimensional surface topography maps of WO_x_ thin films deposited at oxygen flow rates of (**a**) 50, (**b**) 60, (**c**) 70, (**d**) 80, and (**e**) 90 sccm. The surface profiles were measured over an area of 50 μm × 50 μm using a Linnik microscopic interferometer. The color corresponds to the change in surface contour.

Linnik microscopic interferometer measurements [37] showed a systematic increase in surface roughness with oxygen flow rate. Figure 6 shows the root mean square roughness of WO_x_ films deposited under different oxygen flow rates. The RMS surface roughness (Rq) increased from 5.03 ± 0.055 nm at 50 sccm to 6.03 ± 0.045 nm at 90 sccm, while average surface roughness (Ra) increased from 3.48 ± 0.026 to 4.12 ± 0.019 nm. The increase was relatively pronounced between 50 and 60 sccm, followed by a more gradual upward trend. This behavior is consistent with the SEM observations showing increasingly distinct particle boundaries and local surface height variations. Figure 7 shows the three-dimensional surface morphology of WOx thin films deposited in a 50 μm × 50 μm region using a Linnik microinterferometer. The surface profile of WO_x_ thin films was measured using an oxygen flow rate of (a) 50, (b) 60, (c) 70, (d) 80 and (e) 90 sccm, respectively. 

### 3.5. Residual Stress

**Figure 8:**
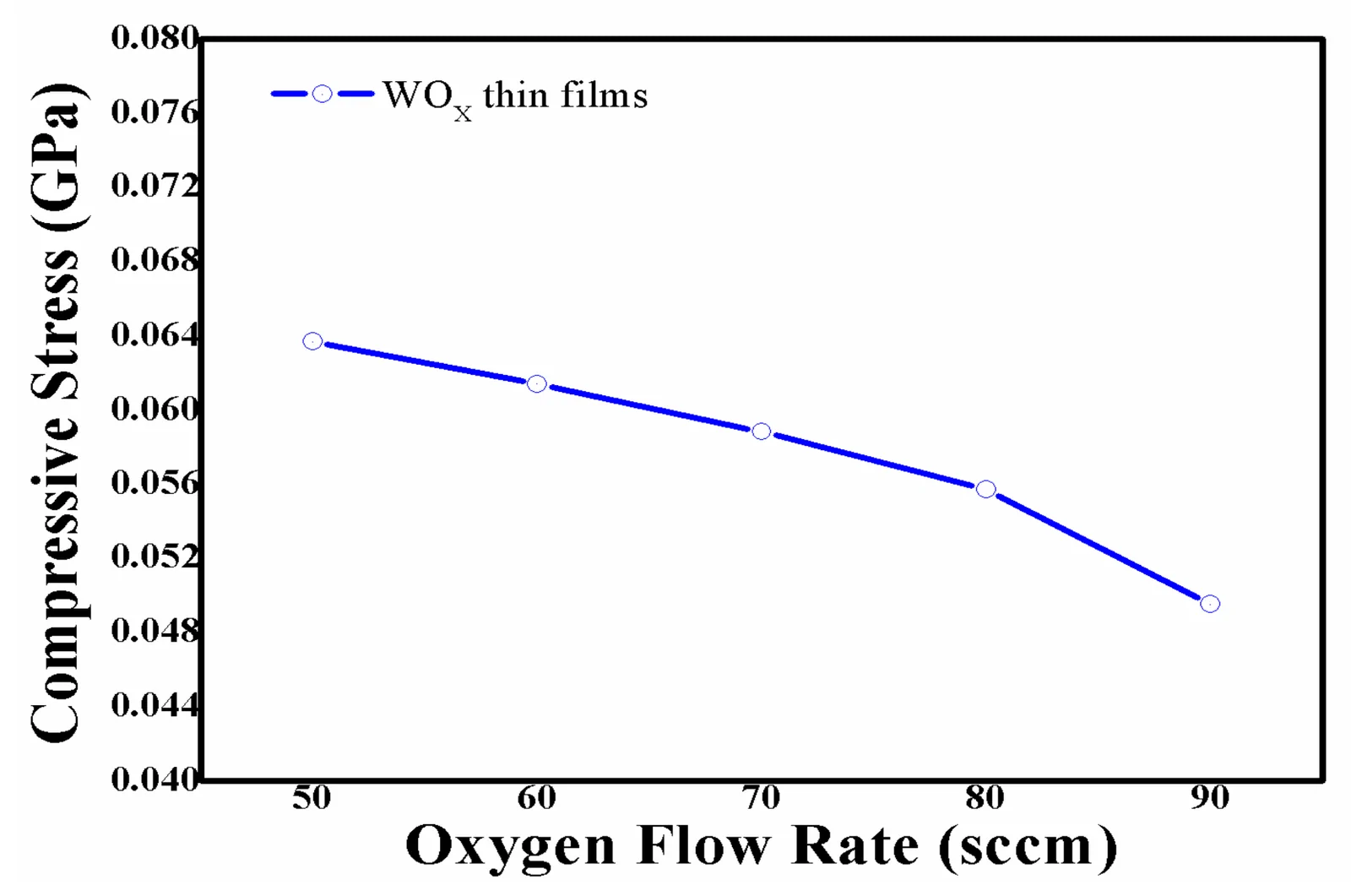
Compressive residual stress in WO_x_ thin films deposited at different oxygen flow rates.

The residual stress of each sample was determined from ten independent Twyman–Green interferometric measurements (repeated interference fringe acquisitions), from which the mean and standard deviation reported in Table 3 were calculated; the uncertainty therefore reflects measurement repeatability rather than a formal propagated error from the Stoney-equation input parameters. All WO_x_ thin films exhibited compressive residual stress. As the oxygen flow rate increased from 50 to 90 sccm, the compressive stress changed from 63.7 to 49.5 MPa. Figure 8 shows the variation trend of compressive residual stress in WO_x_ films deposited under different oxygen flow rates. Although the overlapping uncertainty ranges between adjacent conditions indicate that small pairwise differences should not be overinterpreted, the overall measurement trend shows that the absolute magnitude of the compressive residual stress gradually decreased with increasing oxygen flow rate.

## 4. Discussion

### 4.1. Effect of Oxygen Flow Rate on Optical Behavior

The monotonic decreases in n_650_ and k_650_ were the most pronounced optical changes observed in this study. In oxide thin films, the refractive index can be affected by changes in effective packing density, porosity, chemical composition, and polarizability. Because the actual film thickness did not change monotonically with oxygen flow rate, the continuous decrease in n_650_ should not be attributed solely to a thickness effect. Related reactive-sputtering studies have shown that changes in oxygen partial pressure, oxygen concentration, and working pressure can simultaneously influence the composition, surface structure, and optical constants of tungsten oxide thin films [14,15,16,17,18,19,20]. In particular, the working-gas conditions can alter transport collisions of the depositing species and their energy distribution upon arrival at the substrate [20]. Therefore, a more plausible explanation for the decrease in n_650_ with increasing oxygen flow rate is that the oxygen atmosphere altered particle transport and surface-growth behavior, leading to changes in the effective packing density, pore structure, or composition of the films rather than a simple measured film thickness.

The decrease in k_650_ from 4.2 × 10^−3^ to 3.1 × 10^−3^ indicates a progressively lower extinction coefficient at 650 nm. At the same time, EDS showed an increase in the apparent O/W atomic ratio with oxygen flow rate. Previous reactive-sputtering studies have shown that oxygen ratio and oxygen partial pressure can regulate oxygen defects, W valence states, and sub-band-gap absorption in WO_3−x_ films [15,22,23,38]. The present results are therefore consistent with an oxygen-flow-dependent modification of the oxidation/defect-related optical state. However, because XPS was not performed, the W^6+^/W^5+^ ratio and oxygen-vacancy concentration cannot be directly determined. Accordingly, the decrease in k_650_ and the increase in the EDS-derived oxygen signal are interpreted as a coupled process–optical–composition correlation rather than direct evidence for a quantitative decrease in oxygen-vacancy concentration. This distinction is important because the observed optical trend is experimentally established, whereas its atomic-scale origin remains to be verified by chemical-state characterization.

In contrast, the apparent optical band gap in the visible region E_g_ varied only slightly, between 3.28 and 3.36 eV, and showed no monotonic trend. The 60 sccm sample exhibited the lowest Eg of 3.28 eV. Because Tauc extrapolation is sensitive to the selected linear region and can also be affected by film thickness, defect states, the Urbach tail, and interference effects, the relatively narrow 0.08 eV variation should not be interpreted as evidence of a major change in the fundamental electronic structure. Instead, within the present process window, oxygen flow appears to have a stronger influence on optical loss and film-growth characteristics than on the apparent absorption edge. This distinction between the monotonic evolution of k_650_ and the non-monotonic, weakly varying Eg is one of the most useful materials-level observations of this study.

### 4.2. Correlation Among Surface Morphology, Elemental Composition, and Roughness

The surface analyses indicate that the effect of oxygen flow rate on morphology is reflected mainly in particle boundaries, local height variations, and surface roughness rather than in a large increase in apparent particle size. The apparent particle size increased only from approximately 11 to 14 nm, whereas Rq increased by about 20% (from 5.03 to 6.03 nm). Related sputtering studies have shown that oxygen concentration, oxygen flow rate, working pressure, and discharge mode can alter the surface roughness, particle aggregation, and microstructure of WO_x_ films [15,16,17,20,21,22]. The present combination of SEM and Linnik interferometry suggests that oxygen flow affects not only lateral surface features but also the vertical distribution of surface height. Therefore, the roughness increase is more appropriately associated with changes in particle-packing mode, local nucleation, surface migration, and agglomeration than with simple grain-size growth. Because the SEM-derived values are apparent surface-feature sizes rather than crystallographic grain sizes, they should be interpreted as morphological indicators.

The EDS-derived apparent O/W ratio increased from 2.94 to 3.47 with increasing oxygen flow rate, supporting a relative increase in the oxygen signal. This trend provides an independent compositional indicator that accompanies the systematic decrease in k_650_ and increase in RMS surface roughness Rq. However, the O/W ratio of the high-flow samples exceeded the ideal stoichiometric value of 3 for WO_3_ and therefore cannot be interpreted as an exact stoichiometric composition. Because EDS is semiquantitative for light elements and may be affected by electron-beam interaction depth, substrate contribution, and surface adsorption, the O/W ratio is used only as an apparent relative-composition parameter.

As mentioned above, the O/W ratios modestly exceeding 3.0 are a recurring feature of EDS-based quantification of WOx films in the literature [39,40], generally attributed to standardless quantification bias toward light elements and/or atmospheric moisture adsorption on the porous film surface, rather than to genuine oxygen-rich bulk stoichiometry [2,27,41]. No stoichiometric standard was measured in this study, so the O/W ratios are standard-free and semiquantitative. We therefore treat the reported O/W values as EDS-derived apparent ratios only.

### 4.3. Residual Stress and Process–Property Correlation

The residual-stress results provide mechanical information complementary to the optical and surface analyses. All samples exhibited compressive residual stress, but its absolute magnitude decreased from 63.7 to 49.5 MPa as the oxygen flow rate increased. Thin-film residual stress generally contains intrinsic and thermal contributions [34,35]. Because the same substrate material and a fixed substrate temperature of 100 °C were used for all samples, the baseline thermal-stress conditions were relatively similar. The observed stress evolution is therefore more reasonably discussed in terms of oxygen-flow-dependent changes in intrinsic growth stress, particle transport, surface packing, and interfacial constraint. Studies of tungsten oxide films prepared by HiPIMS and PLD have likewise related composition, density, microstructure, and deposition energy to mechanical properties and residual stress [25,26]. The reduction in the absolute magnitude of compressive stress observed here is consistent with a progressive modification of the film-growth state as oxygen flow increases. However, the present measurements do not by themselves establish a specific atomic-peening or defect-relaxation mechanism, and the stress differences should be interpreted as an overall process-dependent trend rather than as proof of a unique microscopic mechanism.

A coherent process–property map emerges from the combined measurements. When the oxygen flow rate increased from 50 to 90 sccm, the apparent oxygen signal and surface roughness increased, whereas the refractive index, extinction coefficient, and absolute magnitude of compressive residual stress decreased; the apparent optical loss in the visible region remained within a narrow, non-monotonic range. The significance of this combined trend is that the optical, surface, and mechanical responses do not evolve independently. Instead, the oxygen-flow condition acts as a common process variable that simultaneously modifies several measurable film characteristics. Importantly, the monotonic optical-constant trends occur despite a non-monotonic film-thickness response, indicating that the optical changes cannot be explained by thickness variation alone. The present data therefore support a thin film process–property interpretation in which oxygen-flow-dependent changes in the deposition environment influence apparent composition and film-growth state, which are reflected in optical loss, surface topography, and residual stress. This integrated correlation is the principal materials-level contribution of the present study. At the same time, the absence of XRD and XPS data limits direct identification of crystalline phase, W valence state, and oxygen-vacancy concentration; these mechanisms should therefore be regarded as physically plausible interpretations requiring further verification.

From an application-oriented perspective, the higher oxygen-flow samples are particularly interesting because they simultaneously exhibit a lower extinction coefficient, higher surface roughness, and a lower absolute magnitude of compressive stress. Such a combination may be useful when optimizing WO_x_ coatings for optical-fiber sensing, where optical loss and surface interaction can both influence the sensor response. To establish the relationship between the current thin-film process performance and the performance of the loss mode resonance gas sensor developed by our team, future measurements will also be needed on the sensitivity, selectivity, response/recovery time, and long-term stability of the gas sensing device.

These results show that increasing oxygen flow produces a coordinated evolution toward a higher apparent oxygen signal, higher surface roughness, lower visible-region optical loss, and a lower magnitude of compressive residual stress, while the apparent optical loss in the visible region thickness effect remains comparatively stable. This combination suggests that oxygen-flow control can be used not merely to adjust one target property but to define a multidimensional processing window for WO_x_ coatings. In practical terms, the present results identify a trade-off space in which higher oxygen flow favors lower optical loss and lower compressive stress while simultaneously increasing surface roughness. This is relevant to future optical-fiber sensing because surface morphology and optical loss can both contribute to the performance of a functional sensing layer. The novelty of the present work therefore resides in the experimentally demonstrated coupling of optical, surface, compositional, and mechanical responses under a single electron-beam-evaporation process variable, together with the observation that these systematic trends persist even though the actual film thickness does not change monotonically.

## 5. Conclusions

In this work, tungsten oxide thin films were successfully prepared by electron-beam evaporation at 100 °C, and the effects of oxygen flow rates from 50 to 90 sccm on their coupled optical, morphological, element compositional, and mechanical stress characteristics were systematically investigated. The principal finding is that oxygen flow rate provides a common process variable capable of simultaneously tuning several measurable film properties. As the oxygen flow rate increased, the refractive index at a wavelength of 650 nm decreased monotonically from 1.943 to 1.876, while the extinction coefficient decreased from 4.2 × 10^−3^ to 3.1 × 10^−3^. Notably, the actual film thickness varied non-monotonically from 135.77 to 174.10 nm; therefore, the monotonic optical-constant trends cannot be attributed solely to thickness variation. The apparent optical band gapin the visible region remained within 3.28–3.36 eV and showed no monotonic dependence on oxygen flow, indicating comparatively limited modification of the apparent absorption edge within the investigated process window. SEM/EDS and Linnik interferometry showed that the apparent O/W ratio increased from 2.94 to 3.47, the apparent surface-feature size increased from approximately 11 to 14 nm, and RMS surface roughness increased from 5.03 to 6.03 nm. All films exhibited compressive residual stress, with its absolute magnitude decreasing from 63.7 to 49.5 MPa. Collectively, these results establish an experimentally supported process–property map in which increased oxygen flow is associated with higher apparent oxygen signal and surface roughness, together with lower extinction coefficient and lower compressive-stress magnitude. This study therefore contributes a practical materials-design framework for controlling multiple properties of electron-beam-evaporated tungsten oxide films under a common deposition-condition matrix.

## Figures and Tables

**Table 1:** Electron-beam evaporation conditions for the WO_x_ thin films.

Sample No.	O_2_ Flow Rate (sccm)	Deposition Rate (Å/s)	Deposition Temperature (°C)	Working Pressure (Pa)
WO_x_-O50	50	1.0	100	2.2 × 10^−2^
WO_x_-O60	60	1.0	100	2.5 × 10^−2^
WO_x_-O70	70	1.0	100	2.8 × 10^−2^
WO_x_-O80	80	1.0	100	3.1 × 10^−2^
WO_x_-O90	90	1.0	100	3.4 × 10^−2^

**Table 2:** Optical properties and actual thicknesses of WO_x_ thin films deposited at different oxygen flow rates.

O_2_ Flow Rate (sccm)	n_650_	k_650_ (×10^−3^)	Film Thickness (nm)	E_g_ (eV)
50	1.943	4.2	135.8	3.36
60	1.919	3.6	174.1	3.28
70	1.913	3.5	166.7	3.35
80	1.903	3.3	163.3	3.33
90	1.876	3.1	164.4	3.32

**Table 3:** Surface, elemental-composition, and residual-stress results for WO_x_ thin films deposited at different oxygen flow rates.

O_2_ Flow Rate (sccm)	Particle Size (nm)	O/W Ratio x	Rq (nm)	Ra (nm)	Residual Stress (GPa)
50	~11	2.94	5.03 ± 0.055	3.48 ± 0.026	−0.0637 ± 0.014
60	~13	3.05	5.63 ± 0.061	3.88 ± 0.029	−0.0614 ± 0.035
70	~13	3.12	5.76 ± 0.054	3.95 ± 0.026	−0.0588 ± 0.027
80	~14	3.16	5.91 ± 0.056	4.04 ± 0.027	−0.0557 ± 0.009
90	~14	3.47	6.03 ± 0.045	4.12 ± 0.019	−0.0495 ± 0.021

## Data Availability

The original contributions presented in this study are included in the article. Further inquiries can be directed to the corresponding author.
